# Individual Factors in Acculturation: An Overview of Key Dimensions

**DOI:** 10.3390/bs15060827

**Published:** 2025-06-17

**Authors:** Ankica Kosic

**Affiliations:** Faculty of Medicine and Psychology, Sapienza University of Rome, 00185 Roma, Italy; anna.kosic@uniroma1.it

**Keywords:** acculturation, migrants, personality factors

## Abstract

This paper explores the influence of personality and individual factors on the acculturation process, providing a comprehensive overview of key concepts and theoretical frameworks. It organizes these factors into distinct categories, including personality traits, emotional, motivational, and cognitive aspects, identity, and self-concept. By examining how each of these dimensions contributes to the way individuals navigate cultural adaptation, this paper sheds light on the complex and multifaceted nature of acculturation. The insights presented emphasize the importance of understanding individual differences in predicting acculturation outcomes and highlight the role of personal factors in shaping the adaptation process.

## 1. Introduction

While the role of socio-cultural variables in acculturation has been widely explored, comparatively little attention has been given to the influence of individual-level psychological factors, particularly personality traits, on acculturation outcomes. Although some scholars have emphasized the importance of individual differences (e.g., van der Zee & Van Oudenhoven, 2013; Kosic, 2006), empirical studies examining the intersection between personality and specific acculturation strategies (e.g., integration, marginalization) remain limited. Furthermore, most existing research has focused on Western host societies and employed personality assessments developed in Western contexts, raising concerns about their cross-cultural validity (Pernice, 1994; Church & Lonner, 1998).

Acculturation refers to the process by which individuals or groups from one culture come into contact with and adapt to another culture, influencing various aspects of behavior, values, and identity (Berry, 1997). In the literature on acculturation, a distinction has been made between the process (acculturation) and its outcome (adaptation). There are two types of adaptive outcomes: psychological and socio-cultural adaptation/adjustment (Ward, 1996; Ward & Kennedy, 1993; Ward & Rana-Deuba, 1999). The first type refers to psychological well-being and the attainment of personal satisfaction in addition to well-being in the new cultural context, while the second type involves acquiring the social skills and behaviors necessary for successfully navigating daily life.

Deci and Ryan (2008) define psychological well-being as a positive condition that reflects both emotional experiences and an evaluative judgment of one’s life. This concept is typically understood through two main frameworks: The hedonic approach views well-being in terms of emotional and cognitive evaluations of life (Diener et al., 2008). According to this perspective, psychological well-being is characterized by (1) frequent positive emotions, infrequent negative emotions, and (2) high levels of life satisfaction. In contrast, the eudaimonic approach posits that true well-being arises from living in alignment with one’s values and realizing one’s potential. Here, happiness is derived not from pleasure, but from living a meaningful, purposeful, and fulfilling life. Ryff and Singer (2006) conceptualize well-being through six dimensions of personal growth and self-actualization: (1) autonomy—living according to one’s own principles; (2) environmental mastery—effectively managing life’s demands; (3) personal growth—continuous development and realization of potential; (4) positive relationships—having warm, trusting interpersonal connections; (5) purpose in life—having clear goals and a sense of direction; and (6) self-acceptance—a realistic and positive self-view.

Closely related to psychological adaptation is the concept of acculturative stress—the psychological strain experienced by individuals as they adjust to a new cultural environment (Williams & Berry, 1991).

Berry’s (1997) bidimensional model of acculturation—featuring strategies such as integration, assimilation, separation, and marginalization—has long provided a foundational framework for understanding how individuals adapt to new cultural environments. However, recent research has extended this framework by highlighting the important role of individual differences, particularly personality traits like openness to experience and neuroticism, in shaping acculturation outcomes (Ryder et al., 2000; van der Zee & Van Oudenhoven, 2013). These studies suggest that acculturation is not a one-size-fits-all process but is influenced by psychological factors that vary from person to person. Additionally, scholars have increasingly emphasized the two-way relationship between acculturation and mental health. Acculturation strategies such as integration are typically linked to lower levels of stress and depression (Ward & Kennedy, 1994), whereas marginalization is often associated with poorer psychological well-being (J. Schwartz et al., 2010). This body of evidence calls for a more holistic and integrative approach that considers several aspects, such as cultural and social factors, individual traits, identity processes, coping mechanisms, and the broader sociopolitical context. As numerous scholars have noted, even if all migrants were given equal opportunities, their acculturation experiences would still differ due to the interplay of personal characteristics and lived experiences (Allik & Realo, 2019; Benet-Martínez, 2012; Berry et al., 2002; Schmitz & Berry, 2011; Ward, 2001; Zhang et al., 2010). In this paper, the term “migrant” is used broadly to refer to all individuals undergoing acculturation, including immigrants, refugees, asylum seekers, and unaccompanied minors. Migration should not be viewed solely as a response to adverse living conditions; when faced with challenges, individuals may either surrender or, conversely, perceive obstacles as opportunities for growth, thereby enhancing their sense of personal efficacy (Bandura, 1997). This perspective on migration highlights the importance of individual agency—the capacity to actively shape and transform one’s life circumstances (Bandura, 2006).

This theoretical review examines the role of individual factors in acculturation, offering an overview of key concepts and theoretical perspectives. We have organized these factors into meaningful categories: personality traits, emotional aspects, motivational factors, cognitive characteristics, values and beliefs, identity and self-concept, and social dimensions. This approach reflects the multifaceted nature of human behavior and allows for a nuanced exploration of how different psychological domains interact with acculturative processes. Each category captures a distinct yet interconnected aspect of the individual that has been shown to influence adaptation to new cultural environments. By organizing the literature in this way, the review not only facilitates conceptual clarity but also supports the identification of specific pathways through which individual differences affect both psychological and socio-cultural adaptation.

We conducted a systematic literature search using the PsychINFO, PsycArticles, and Web of Science databases for articles published up to now. The thematic filters we used were (a) Migration: migrants OR immigrants OR ‘ethnic groups’ OR emigration and immigration OR ‘Forced migrants’ OR refugee* OR ‘asylum seeker*’; (b) ‘Personality factors’ OR ‘Individual factors’ OR ‘Personal dimensions’ OR ‘Motivations’ OR ‘Emotions’ OR ‘Cognitive factors’ OR ‘Identity’; and (c) ‘Acculturation’ OR ‘Adaptation’. The results of these searches, however, excluded relevant articles and book chapters of which we were aware, so we also used Google Scholar to extend the search, using the same filters and period.

## 2. The Role of Personality in the Acculturation Process

Personality refers to the stable set of psychological traits and behavioral patterns that influence an individual’s thoughts, emotions, and actions across various situations. While acculturation has been extensively studied, research exploring its relationship with personality and the self remains comparatively limited. Several key issues emerge when examining the influence of personality on acculturation.

One major debate concerns the universality versus cultural specificity of personality traits. This debate reflects a broader issue in cross-cultural psychology: whether behavior is primarily driven by inherent dispositions or shaped by situational and environmental factors. Three primary perspectives have emerged. Relativists argue that personality constructs vary significantly across cultures (Markus & Kitayama, 1998), absolutists claim that traits are universal and independent of cultural context (Eysenck, 1983), and universalists suggest that while core traits exist across cultures, their development and expression are influenced by cultural factors (Berry et al., 2002). McCrae and Costa (1997), for example, assert that all cultures permit the expression of fundamental individual differences, although cultural environments may shape how these traits are encouraged or expressed.

Another concern is whether personality assessments developed in Western contexts are applicable to individuals from diverse cultural backgrounds, particularly immigrants. Pernice (1994) and others have questioned the cross-cultural validity of these measures. Church and Lonner (1998) suggest that using an etic approach—applying an existing instrument across cultures—yields more comparable results than developing culture-specific tools (emic approach).

Despite such theoretical debates, researchers widely agree that personality influences how individuals perceive and adapt to cultural differences. Traits such as sociability, emotional regulation, and openness significantly affect one’s capacity to integrate into a new culture (van der Zee & Van Oudenhoven, 2013, 2014).

The following sections explore how specific personality traits are linked to acculturation strategies, psychological and socio-cultural adaptation, and acculturative stress.

### 2.1. The Big Five Personality Traits and Acculturation

One of the most widely recognized personality models is the Big Five, also known as the Five-Factor Model (Costa & McCrae, 1992; Digman, 1990; Goldberg, 1993). This framework describes personality through five broad dimensions: (1) extraversion (sociability and energy), (2) agreeableness (warmth and cooperation), (3) conscientiousness (organization and self-discipline), (4) neuroticism (emotional instability and anxiety), and (5) openness to experience (curiosity and intellectual engagement).

Numerous studies have shown that traits such as extraversion, agreeableness, conscientiousness, emotional stability (low neuroticism), and openness are strong predictors of positive acculturation outcomes (Bakker et al., 2004; Caligiuri, 2000; Swagler & Jome, 2005; Ward et al., 2004; for a review, see van der Zee et al., 2016).

Extraversion fosters social engagement, helping individuals form new relationships in host communities. It is positively associated with the development of social networks and the integration acculturation strategy (Sam & Berry, 2006).

Emotional stability allows individuals to manage stress, reduce anxiety, and cope effectively with the uncertainties of acculturation. It acts as a protective factor against acculturative stress (Zhou et al., 2008). In contrast, individuals high in neuroticism often struggle with acculturative stress and are more likely to experience negative psychological outcomes (J. Schwartz et al., 2010; Schmitz & Berry, 2011).

Openness to experience facilitates curiosity, learning, and engagement with unfamiliar cultural practices. It aligns with the integration strategy and promotes cognitive flexibility, which supports psychological well-being and socio-cultural adaptation (McCrae & Costa, 1997; Piontkowski et al., 2000; Sam & Berry, 2006; Van Oudenhoven et al., 2003; Ward et al., 2001).

Similarly, agreeableness supports inter-cultural communication and helps maintain harmonious social relationships.

Conscientiousness enhances goal-setting, self-regulation, and adaptation to new social and economic environments, which are vital for successful acculturation, particularly for achieving adaptation-related goals. Conscientious individuals tend to be more proactive in learning the host language, understanding cultural norms, and managing the practical demands of life in a new country. Their disciplined and goal-driven nature helps them navigate essential aspects of socio-cultural adaptation, such as securing employment, finding housing, and handling financial responsibilities (Sam & Berry, 2006).

These traits are generally negatively correlated with an immigrant’s desire to return to their country of origin, indicating greater long-term adaptation (Caligiuri, 2000). Conversely, high levels of neuroticism and low levels of other Big Five traits are often associated with psychological distress and social isolation (J. Schwartz et al., 2010).

In summary, different acculturation strategies—integration, assimilation, separation, and marginalization—are associated with distinct personality profiles. Integration is positively linked with extraversion, emotional stability, sociability, openness, agreeableness, and conscientiousness, and negatively correlated with neuroticism, psychoticism, and aggression–hostility (Chen et al., 2008; Ryder et al., 2000; see also for a review Schmitz & Schmitz, 2022; van der Zee et al., 2016). Marginalization is associated with higher neuroticism, lower conscientiousness and extraversion, and greater impulsivity and hostility. Assimilation has mixed associations: it correlates with sociability and agreeableness but also with neuroticism and anxiety (Luijters et al., 2006). Separation correlates positively with neuroticism, sensation-seeking, and aggressiveness, and negatively with extraversion and self-assurance.

These findings suggest that individuals with traits conducive to openness, stability, and social initiative are more likely to pursue integration, whereas those high in neuroticism and aggression may gravitate toward marginalization or separation.

However, a key limitation of the Big Five model in inter-cultural contexts is that its broad traits may not fully reflect the specific behaviors and culture-sensitive skills—such as learning language or building inter-cultural relationships—that are crucial for successful adaptation (van der Zee & Van Oudenhoven, 2013; Ward et al., 2004).

### 2.2. Inter-Cultural Traits and Acculturation

van der Zee and Van Oudenhoven (2000, 2001) proposed five inter-cultural traits particularly relevant in inter-cultural contexts: emotional stability (staying calm in unfamiliar or stressful situations), social initiative (actively engaging in social interactions), open-mindedness (accepting different cultural norms and values), cultural empathy (understanding and sharing others’ emotions and experiences), and flexibility (adapting behavior in changing environments). These traits are measured using the Multi-Cultural Personality Questionnaire (MPQ).

While emotional stability, social initiative, and open-mindedness overlap with Big Five dimensions, cultural empathy and flexibility are more specific to inter-cultural contexts. Research shows that these traits predict positive acculturation outcomes among migrants, expatriates, and their families (e.g., van der Zee et al., 2002; Ali et al., 2003; Peltokorpi, 2008; for a review, see van der Zee et al., 2016).

### 2.3. Meta-Traits and Acculturation

Beyond the Big Five and inter-cultural traits, meta-traits such as core self-evaluations play a central role in acculturation. These evaluations represent how individuals view their own worth, competence, and control over life circumstances. Core self-evaluations consist of four key lower-order traits: self-esteem, generalized self-efficacy, locus of control, and emotional stability (Judge et al., 2002, 2003). High core self-evaluations are associated with psychological resilience and adaptability, while low evaluations correlate with distress and maladjustment.

Self-awareness: Positive self-perceptions act as psychological resources that help individuals manage stress and overcome setbacks, whereas negative self-perceptions are often linked to maladjustment, depression, and other psychological difficulties. Self-awareness is inherently both evaluative and motivational—people typically feel discomfort when they fail to meet their own standards or expectations. In response, they may either work to improve themselves to align with these standards or attempt to escape the distress associated with self-awareness (Csikszentmihalyi & Figurski, 1982). In the context of immigration, a healthy level of self-awareness can facilitate adaptation, and excessive self-orientation may contribute to psychological distress (Furnham & Bochner, 1986).

Excessive self-focus and heightened cultural self-awareness have been associated with depression and low self-esteem (Furnham & Bochner, 1986). This highlights the complex role of self-reflection in acculturation, suggesting that self-esteem is a strong predictor of adaptation within immigrant populations (Valentine, 2001). Research by Sam and Virta (2003) revealed that individuals with a bicultural or integrated attitude had higher levels of self-esteem. Self-esteem is a protective factor against prejudice and downward mobility (Sam & Virta, 2003). It helps immigrants reframe challenging experiences in ways that preserve agency and optimism (Kosic & Triandafyllidou, 2003).

Self-efficacy refers to an individual’s belief in their ability to successfully accomplish tasks, overcome challenges, and reach goals in specific situations (Bandura, 1977). It is essential for motivation, learning, and behavior, influencing how people approach obstacles, persist through adversity, and remain resilient in the face of setbacks. Self-efficacy enhances motivation and problem-solving coping and is linked positively to all acculturation strategies—except marginalization, with which it is negatively associated (Ramdhonee & Bhowon, 2012; Schwarzer & Warner, 2013). Refugees are more likely to experience a reduced sense of self-efficacy compared to voluntary immigrants, especially during the initial months after arriving in the host country. This can lead to heightened perceptions of dependency on external factors and diminish motivation for learning and acculturation (Zimmerman, 2000).

Locus of control: Several studies suggest that migrants with an internal locus of control, who believe they have personal control over life events, report lower levels of acculturative stress (while an external locus of control is linked to psychological distress) (Van Selm et al., 1997; Ward & Kennedy, 1992, 1994).

In conclusion, although extensive research has explored the relationship between personality traits and acculturation, the findings remain somewhat inconsistent, with strong consensus emerging for only a few dimensions (e.g., neuroticism).

While numerous studies have identified extraversion as a generally beneficial trait in the acculturation process, its effectiveness is not universal across all cultural contexts. Extraversion, characterized by sociability, assertiveness, and high energy, has been positively associated with social integration and psychological well-being in many Western, individualist societies, where outgoing behavior is valued and encouraged (Sam & Berry, 2006; Ward et al., 2004). In such environments, extraverted individuals are often more successful at forming social networks and engaging in the host culture, which supports adaptive outcomes such as reduced loneliness, increased social support, and enhanced psychological resilience.

However, these benefits may not translate seamlessly into collectivist or high-context cultures, where interpersonal restraint, humility, and indirect communication are more normative. In such cultural settings, extraverted behaviors may be perceived as intrusive or socially inappropriate, potentially leading to interpersonal tension, exclusion, or miscommunication. This mismatch between individual personality traits and host cultural expectations has been documented in several studies. For example, Ward and Chang (1997) found that American expatriates in Singapore—who were more extraverted than the local population—experienced increased social rejection and frustration due to their tendency to initiate frequent and intense social contact, which was not reciprocated or welcomed in the same manner. This highlights a central critique of the assumption that extraversion is universally adaptive.

This divergence in outcomes can be better understood through Searle and Ward’s (1990) cultural fit hypothesis, which proposes that successful acculturation depends not solely on an individual’s traits but on how well these traits align with the social norms and values of the host culture. In other words, the psychological benefits of extraversion—or any trait—are context-dependent. The cultural fit model suggests that traits conducive to adaptation in one cultural environment may become maladaptive in another. It emphasizes the interactive nature of personality and cultural context, offering a more nuanced framework than trait-based models alone. From this perspective, extraversion can be seen as a “double-edged sword”: in extraversion-valuing societies (e.g., the United States, Canada, and Australia), the trait facilitates smoother integration and personal satisfaction. But in cultures that prize group harmony, modesty, and indirectness (e.g., Japan, Korea, and Singapore), overt extraversion may hinder inter-cultural relationships, evoke resistance, or exacerbate acculturative stress. This underscores the importance of trait-context congruence, not merely the trait itself.

Further complicating this picture is the distinction between trait expression and trait perception. Even if extraverted individuals attempt to regulate their behavior to align with cultural expectations, their motivations and internal inclinations might still cause psychological discomfort or conflict. Thus, cultural fit affects not only external adaptation (e.g., social success) but also internal outcomes, such as identity coherence and emotional well-being.

In sum, while personality traits like extraversion can offer valuable insights into acculturation strategies and outcomes, they must be considered in light of the host society’s cultural norms. The effectiveness of a trait is not absolute, but moderated by the cultural environment, supporting a more dynamic, context-sensitive view of personality’s role in inter-cultural adaptation. This underscores the need for future research to explore personality–context interactions, rather than relying on static, trait-centric models that overlook the social ecology of acculturation. Addressing these issues can provide a more nuanced understanding of acculturation as a dynamic interplay between individual agency and contextual factors.

Furthermore, the majority of research on personality and acculturation has predominantly focused on the Big Five personality traits, often overlooking other important psychological dimensions such as persistence, optimism, and hope. These traits, while less commonly examined, may serve as critical internal resources that help migrants cope with the complex and often stressful demands of adjusting to a new cultural environment (Cobb et al., 2019). Traits like optimism and hope can foster resilience, enhance motivation, and support a proactive orientation toward challenges, thereby potentially influencing both short- and long-term adaptation outcomes. Moreover, much of the existing literature remains centered on traditional frameworks—particularly theories of acculturation strategies (e.g., integration, assimilation, separation, and marginalization) and psychological adaptation—while giving comparatively little attention to more-contemporary and multidimensional models of psychological well-being, such as the eudaimonic approach articulated by Ryff and Singer (2006). These models emphasize not just the absence of psychological distress, but the presence of positive functioning, self-realization, and meaningful engagement with life. Incorporating such perspectives may yield a more holistic understanding of migrant well-being, moving beyond mere cultural adjustment to include broader indicators of human flourishing and psychological resilience.

## 3. Motivations and Acculturation

Motivation is a fundamental psychological construct that underpins human behavior, shaping how individuals engage with their environment, make decisions, and pursue goals. In the context of migration, motivation serves as a critical driver of both the decision to emigrate and the strategies adopted during acculturation process. The interplay between motivational orientation and acculturation outcomes is multifaceted, yet often underexplored in mainstream migration literature.

One of the earliest and most influential frameworks in this area is McClelland’s (1987) trichotomy of motivational needs, which includes the need for achievement, power, and affiliation. Building on this, Boneva and Frieze (2001) found that individuals who choose to emigrate often exhibit higher achievement as well as power motives and lower affiliation needs compared to those who remain in their home countries. This suggests that migrants are frequently motivated by aspirations for personal advancement, recognition, and autonomy—goals often aligned with self-enhancement rather than social conformity. High achievers may seek new environments that offer greater challenges and opportunities, while those driven by power motivation may embrace the risks and uncertainties of migration as a path to status and influence.

However, this framework is not without its limitations. It risks overgeneralizing migrant motivation as being primarily individualistic and goal-driven, which may not accurately reflect the complex, context-bound decisions migrants make—particularly in forced or constrained migration scenarios (e.g., refugees, asylum seekers). Moreover, low affiliation motivation among migrants could be misinterpreted as social disengagement, rather than a temporary coping strategy or a reaction to pre-migration conditions.

Offering a broader perspective, Tartakovsky and Schwartz (2001) proposed a tripartite model of migration motives: preservation, self-development, and materialism. This framework recognizes the diversity of motivational drivers—ranging from the need for security and psychological stability to the pursuit of personal growth and financial gain. Unlike McClelland’s model, this approach acknowledges both protective (defensive) and promotive (aspirational) motivations. Critically, while this model incorporates more nuance, it still tends to treat motives as relatively static rather than dynamic forces that evolve during the migration and settlement process. Furthermore, it does not fully account for the role of social context, such as discrimination, legal barriers, or economic systems, which often shape the feasibility and outcomes of migrants’ motivational goals.

From a self-determination theory perspective, Deci and Ryan (1985) distinguished between intrinsic and extrinsic motivation, offering another valuable lens for understanding acculturation. Intrinsic motivation—engaging in activities for personal fulfillment and internal satisfaction—has been associated with more-successful and psychologically adaptive acculturation outcomes (Berry, 1997). Research by Chirkov et al. (2003) demonstrated that migrants with intrinsic motivation are more likely to develop bi-cultural identities, build meaningful relationships with host communities, and experience higher psychological well-being. In contrast, extrinsically motivated individuals, driven by external rewards (e.g., social approval, job security), may engage in surface-level cultural adaptation without internalizing new cultural values. While extrinsic motivation may support short-term functional goals (e.g., learning the language for employment), it is less likely to foster long-term emotional or cognitive integration.

Nevertheless, this dichotomy between intrinsic and extrinsic motivation can sometimes oversimplify the complex interplay between personal agency and structural constraints. Migrants often operate within mixed motivational frameworks, where intrinsic and extrinsic motives coexist or shift depending on context. For instance, an immigrant may begin with extrinsic goals (e.g., financial stability) but later develop intrinsic motivation as they build new social bonds or find meaning in their experiences abroad.

The need for cognitive closure (NCC; (Kruglanski, 1989))—a preference for certainty and an aversion to ambiguity—has also been implicated in shaping acculturation outcomes. Some studies found a positive correlation between NCC and acculturative stress, particularly when migrants face unfamiliar or unpredictable environments (Kosic, 2002b). Kosic et al. (2004) further investigated the role of NCC in immigrants’ acculturation experiences. Three studies have provided evidence that immigrants’ acculturation to a host culture is shaped by an interaction between their NCC (Kruglanski & Webster, 1996) and the type of reference group they form upon arrival. When immigrants establish close social ties primarily with co-ethnics, a higher NCC is associated with a stronger attachment to their culture of origin and a reduced inclination to assimilate into the host culture. Conversely, when immigrants form close relationships with members of the host society, a high NCC predicts a greater tendency to adapt to the host culture and a weaker adherence to their culture of origin. This suggests that the social context in which migrants situate themselves plays a moderating role in how cognitive–motivational dispositions influence acculturation outcomes (Kosic et al., 2004; Ramelli et al., 2013). These findings suggest that NCC interacts not only with individual psychological traits but also with the availability and quality of social networks, highlighting the importance of contextual and interpersonal factors in acculturation. Florack et al. (2014) confirmed that initial cross-group friendships reduce anxiety and increase self-confidence in communication, which in turn enhances positive attitudes and further cross-group contact. These findings underscore the importance of facilitating opportunities for interaction and support between immigrants and host society members during the early stages of immigration. In this critical phase, cross-group friendships can help alleviate anxiety and uncertainty in communication, allowing immigrants to engage more comfortably with the mainstream group.

Similarly, regulatory focus theory provides additional insight into motivational orientations. Migrants with a prevention focus, who are concerned with avoiding negative outcomes, tend to experience more regret, particularly when exposed to upward social comparisons with co-nationals or perceived discrimination in the host context (Kosic et al., 2024). This contrasts with migrants who adopt a promotion focus, motivated by growth and advancement, and who typically report more positive adaptation outcomes. The interaction between regulatory focus and environmental feedback (e.g., social comparisons, cultural acceptance) illustrates how motivational orientation is not just an internal trait, but one that is shaped—and potentially constrained—by external perceptions and experiences.

The need for approval and the capacity for self-monitoring also play crucial roles in how migrants navigate social dynamics in the host culture. Self-monitoring, as conceptualized by Snyder (1974), reflects the ability to regulate one’s behavior in social contexts. High self-monitors, particularly those high in the “getting-ahead” dimension (Lennox & Wolfe, 1984), are often adept at interpreting social cues and adjusting their behavior accordingly, facilitating smoother social integration and higher expatriate performance (Caligiuri & Day, 2000). Conversely, those high in “getting-along” may prioritize avoiding social disapproval, which can be linked to social anxiety and a preference for separation strategies—maintaining strong cultural ties while disengaging from the host society (Kosic, 2002a; Kosic et al., 2006). While high self-monitoring may support successful adaptation, it also raises questions about authenticity and psychological strain, particularly when the individual’s values conflict with those of the host society.

In conclusion, motivation is a multifaceted and dynamic force in the acculturation process, influencing not only the decision to migrate but also how individuals navigate cultural transitions, build relationships, and construct their identities. While intrinsic motivation, promotion focus, and adaptive self-monitoring are generally associated with more favorable adaptation outcomes, these effects are not uniform and are highly context-dependent. Psychological constructs like the need for cognitive closure and need for approval further complicate the picture, as they interact with external social conditions and internal regulatory mechanisms.

Critically, much of the literature still tends to approach motivation from an individualistic perspective, often neglecting the structural and contextual factors—such as racism, legal status, and socioeconomic inequality—that constrain migrants’ agency and shape their motivational trajectories. Future research should adopt more ecological and intersectional frameworks that account for how motivation interacts with broader social, cultural, and political forces. Integrating models of psychological well-being (e.g., Ryff & Singer, 2006) into migration studies can also enrich our understanding of what constitutes successful adaptation—not merely survival or economic integration, but flourishing and fulfillment in a new cultural context.

## 4. Emotional Characteristics and Acculturation

Migrants often face a complex interplay of stressors that can lead to heightened anxiety, psychological suffering, and, in some cases, post-traumatic stress disorder (PTSD). The migration process frequently involves exposure to traumatic events such as war, persecution, forced displacement, or dangerous journeys, which can leave enduring psychological scars. Even after arrival in a new country, migrants may encounter chronic stress due to language barriers, discrimination, loss of social status, financial insecurity, and cultural dislocation. These cumulative stressors can significantly impair mental health, particularly when access to social support or mental health services is limited. For refugees and asylum seekers, the risk of PTSD is especially high, as their trauma is often compounded by prolonged uncertainty about legal status and fears of deportation. Research suggests that untreated trauma can impede adaptation, erode resilience, and result in long-term emotional suffering, underscoring the urgent need for trauma-informed care and culturally sensitive mental health interventions in migrant populations.

Homesickness, often trivialized in mainstream discourse, deserves recognition as a significant emotional hurdle in the acculturation journey. It encompasses not just nostalgia but a deeper existential dislocation, as noted in R. K. Papadopoulos (2015) concept of “nostalgic disorientation.” This framework emphasizes the loss of umwelt—the psychosocial environment that anchors identity and meaning. Tarantini (2014) adds that the experience of “home” serves as a cognitive and emotional schema that helps migrants process past experiences and project future aspirations. While often treated as a transient phenomenon, homesickness can become chronic and debilitating, particularly in forced migration contexts. Despite this, research in this domain remains sparse and overly descriptive. More empirical work is needed to explore how homesickness interacts with variables like age, gender, and cultural distance, and whether interventions targeting meaning-making can mitigate its effects.

According to Berry’s Acculturation Model, migrants experience stress as they navigate between maintaining their original cultural identity and adapting to a host culture. This model outlines four acculturation strategies—integration, assimilation, separation, and marginalization—with each carrying different levels of psychological risk. For instance, marginalization, where individuals feel disconnected from both their original and host cultures, is often associated with the highest levels of stress and poor mental health outcomes, including anxiety and PTSD.

Additionally, the Stress-Coping Framework helps explain how migrants respond to the pressures of relocation and trauma. According to this model, when the demands of migration (e.g., loss, instability, and threat) exceed coping resources, individuals may experience chronic stress and mental health decline. If traumatic events are involved, especially without adequate processing or support, the risk of developing post-traumatic stress disorder increases significantly.

Coping strategies represent one of the most thoroughly studied psychological dimensions of acculturation. Rooted in the transactional model of stress (Lazarus & Folkman, 1984), coping involves cognitive and behavioral efforts to manage perceived stressors. These are typically categorized into problem-focused, emotion-focused, and avoidance strategies (Endler & Parker, 1999), though other typologies also exist. Empirical research consistently shows that problem-focused coping—such as seeking employment or learning the host language—is associated with better acculturative outcomes, including improved mental health and social integration (Crockett et al., 2007; Noh & Kaspar, 2003). In contrast, avoidance strategies, such as social withdrawal, are linked with elevated psychological distress and poor adaptation (Berry, 1997; Carver, 1997). Nevertheless, passive strategies like acceptance or emotional compromise may offer short-term relief, especially in environments where agency is constrained (D. F. K. Wong, 2002). The cultural context critically shapes coping preferences (Kuo, 2011, 2012). For example, individuals from collectivistic societies may favor emotion-focused or social support-based coping to preserve group harmony, whereas those from individualistic cultures are more likely to adopt problem-solving approaches (Kuo, 2014). Chun et al., 2006). This implies that interventions promoting problem-focused coping must be culturally attuned; a universalist approach risks overlooking deeply ingrained cultural scripts regarding stress and agency.

Research also links coping strategies to acculturation strategies (Schmitz, 1992; for a review see Schmitz, 2001; Schmitz & Schmitz, 2022). Integration is positively correlated with task-oriented coping, whereas separation tends to co-occur with avoidance. Notably, assimilation incorporates both task- and emotion-oriented coping. These associations underline the functional role of coping in shaping—and being shaped by—acculturative choices. However, the relationship is likely bidirectional. Maladaptive coping may lead to marginalization, but marginalization itself may reduce access to coping resources, creating a negative feedback loop. Moreover, coping strategies are not fixed; they evolve over time and across contexts (Aldwin, 2007; P. T. P. Wong & Wong, 2006). Longitudinal studies that map these shifts are needed to better understand the temporal dynamics of acculturative coping.

Emotional intelligence (EI) has emerged as a key psychological construct in understanding individual differences in adaptation and acculturation. It is generally conceptualized along two dimensions: trait emotional intelligence and ability emotional intelligence (Petrides & Furnham, 2000, 2001). Ability EI refers to a set of cognitive–emotional abilities assessed through performance-based tests, akin to IQ assessments, while trait EI comprises self-perceived emotional abilities and is measured through self-report instruments. This trait-based conceptualization includes components such as emotional awareness, regulation, and empathy, which are closely linked to personality factors. Trait EI has shown significant associations with broader personality dimensions; for example, it correlates negatively with neuroticism and positively with extraversion (Petrides & Furnham, 2001; van der Zee et al., 2002). These personality linkages suggest that emotionally intelligent individuals are more likely to exhibit adaptive social behaviors and emotional resilience—qualities essential for successful acculturation. Further supporting this view, trait EI is positively associated with cultural empathy and social initiative, key constructs within inter-cultural competence frameworks (Ponterotto et al., 2011). These traits enable migrants to navigate the interpersonal demands of a multi-cultural environment. Importantly, studies like Lopez-Zafra and El Ghoudani (2014) found that Moroccan female migrants in Spain who preferred integration strategies had significantly higher EI than those who favored assimilation, separation, or marginalization. This suggests that EI may predispose individuals toward more adaptive acculturation strategies, such as integration. However, findings from Schmitz and Schmitz (2022) complicate this picture. Their meta-analysis revealed that both integration and assimilation were positively associated with higher EI, while marginalization was negatively related. Surprisingly, separation showed inconsistent associations. This complexity highlights a limitation of existing research: it tends to treat acculturation strategies as static or mutually exclusive, when, in reality, migrants may employ hybrid or shifting strategies depending on context. Thus, while trait EI supports acculturative competence, its relationship with specific strategies may be moderated by situational and cultural factors that require further investigation.

Resilience plays a pivotal role in how migrants navigate the psychological and social challenges of migration, such as discrimination, language barriers, and social isolation. Defined as the capacity to maintain or regain psychological well-being in the face of adversity, resilience enables individuals to adapt to significant stressors while maintaining functional and emotional stability (Bonanno, 2005; Luthar et al., 2000; R. Papadopoulos, 2007; Pooley & Cohen, 2010). Scholars increasingly view resilience not as a static trait but as a dynamic process involving the interaction of internal capacities and external resources (Connor & Davidson, 2003; Kosic, 2021; Rutter, 1990). Resilient migrants are more likely to use proactive coping strategies, regulate their emotions effectively, and engage in problem-solving, all of which support their mental health and socio-cultural adaptation (Aspinwall & Taylor, 1997; J. Schwartz et al., 2010). According to Iacoviello and Charney (2014), six psychosocial factors are central to fostering resilience: optimism, active coping skills, cognitive flexibility, supportive social relationships, physical well-being, and a personal moral compass. Spirituality and religious beliefs are also increasingly recognized as vital sources of strength (Manning, 2013). Notably, resilience is not incompatible with psychological symptoms. As R. Papadopoulos (2007) argues, individuals may simultaneously experience distress and display resilient traits, such as humor, caregiving, or maintaining purpose. This duality challenges deficit-based approaches and emphasizes the importance of identifying and nurturing strengths even amid suffering. Harvey (2007) similarly contends that vulnerability and resilience coexist within all individuals, reinforcing the need for nuanced understandings of adaptation.

Longitudinal studies confirm resilience’s positive influence on life satisfaction and effective coping throughout the acculturation process (Chun et al., 2006). As such, resilience functions as a protective factor that reduces acculturative stress and supports a smoother transition in a new cultural context.

Sense of Coherence: The Sense of Coherence (SOC), a central concept in Antonovsky’s (1979, 1987) Salutogenic Theory, refers to an individual’s capacity to view life as structured, manageable, and meaningful. It comprises three components: comprehensibility (perceiving life events as ordered and predictable), manageability (believing one has the resources to cope), and meaningfulness (finding life challenges worthy of engagement). An SOC is strongly associated with positive health outcomes and psychological resilience among migrant populations (Jibeen & Khalid, 2010; Pallant & Lae, 2002). In migration contexts, an SOC enables individuals to interpret and respond to the stressors of cultural transition, such as language difficulties, discrimination, or identity conflicts. A strong SOC enhances psychological adaptation by promoting a proactive, resource-based approach to life challenges (McSherry & Holm, 1994; Daniel & Ottemöller, 2022). Antonovsky’s framework marks a shift from deficit-focused models by emphasizing generalized resistance resources (GRRs)—such as social networks, cultural identity, and institutional access—that reinforce an SOC and buffer the impact of stress. This salutogenic approach encourages strength-based interventions that promote an SOC and thereby improve long-term migrant well-being.

Religiosity and spirituality: Religiosity and spirituality are critical psychosocial resources that influence migrants’ adaptation experiences. While religiosity often refers to institutionalized belief systems, rituals, and affiliations, spirituality is more personal, reflecting an individual’s search for meaning, purpose, and transcendence (George et al., 2002). Both constructs can provide psychological stability and social support during resettlement. Religious coping strategies—classified as positive or negative—differentially affect adaptation. Positive religious coping, such as prayer, community worship, and trust in a benevolent deity, has been shown to foster hope and emotional regulation under stress. Conversely, negative coping (e.g., religious discontent, guilt, or punitive views of God) may exacerbate psychological distress (Ali et al., 2003; George et al., 2002).

For many migrants, engagement with religious communities facilitates emotional support, practical aid, and a sense of belonging—enhancing both psychological and socio-cultural adaptation (S. J. Schwartz et al., 2011). However, the role of religiosity is context-dependent. In some cases, a strong religious identity supports integration; in others, it may contribute to separation from the host society, particularly when value systems clash (Phalet & Güngör, 2004).

Research on the structural outcomes of religiosity—such as employment, education, and income—yields mixed results (Khattab, 2009; Kogan et al., 2020). While religious engagement may bolster social capital and motivation in some cases, in others, it may limit adaptation due to conflicting cultural expectations or institutional discrimination. Moreover, the protective value of religious coping is not uniform; some studies report reduced distress through religious practice (Sanchez et al., 2012), while others find minimal effects (Dunn & O’Brien, 2009). This variability underscores the need for culturally and contextually sensitive research that considers the diverse expressions of faith among migrant populations.

Attachment styles: Attachment theory, developed by Bowlby (1982), posits that early relationships with caregivers shape internal working models that influence how individuals relate to others and respond to stress throughout life. In adulthood, these attachment styles play a key role in shaping migrants’ adjustment to new cultural environments. Attachment styles are generally categorized into four types (Bartholomew & Horowitz, 1991): (a) Secure—positive view of self and others, comfort with intimacy and autonomy; (b) Preoccupied—negative view of self and positive view of others, anxious and approval-seeking; (c) Dismissive—positive view of self and negative view of others, emotionally distant and self-reliant; and (d) Fearful—negative view of self and others, socially avoidant and emotionally conflicted.

These dimensions closely align with Berry’s (1997) acculturation strategies. Secure attachment is associated with the integration strategy, enabling individuals to maintain strong ties to both their heritage and host cultures. In contrast, dismissive or fearful attachment styles may lead to separation or marginalization, impeding social adaptation (Polek et al., 2008; Van Oudenhoven & Hofstra, 2006).

Empirical studies among migrants show that secure attachment correlates with better psychological outcomes, though not always with socio-cultural adjustment (Polek et al., 2010). For instance, Wang and Mallinckrodt (2006) found that anxious attachment predicted lower levels of integration and increased the likelihood of adopting assimilation or avoidance strategies—often resulting in poorer psychological well-being. The migration process itself often disrupts attachment systems, especially for unaccompanied minors or those leaving family behind. The “family mandate” (Gozzoli & Regalia, 2005)—expectations placed on migrants to fulfill familial duties—can become a source of emotional strain if unmet. Adolescents may struggle to navigate these obligations in unfamiliar environments, increasing psychological distress (Scabini & Donati, 1993).

Attachment styles also interact with other psychological traits, such as core self-evaluations and coping mechanisms. Securely attached individuals tend to employ active coping, enhancing adaptation, while insecure individuals may resort to emotion-focused or avoidant strategies (Judge et al., 2002), which hinder acculturation.

## 5. Cognitive Characteristics and Acculturation

Cognitive styles: Cognitive styles are among the earliest psychological constructs examined in the context of acculturation, offering insight into how individuals process, interpret, and respond to cultural change. Two central cognitive styles—field dependence/independence (Witkin et al., 1954) and dogmatism (Rokeach, 1960)—have been instrumental in understanding psychological differentiation and adaptation (Berry, 1971; Witkin & Berry, 1975). Field independence refers to a cognitive tendency to rely on internal frames of reference and to separate information from its contextual background. In contrast, field-dependent individuals are more influenced by external cues and tend to perceive their environment holistically. According to Berry (1976), field-independent individuals are generally better equipped to cope with cultural transitions, as they are less reliant on social cues and more able to preserve a stable self-concept amidst change. Witkin and Berry (1975) observed that field-independent individuals experience less acculturative stress and demonstrate a greater capacity for psychological differentiation, which can facilitate adaptive responses to unfamiliar cultural settings. Conversely, field-dependent individuals may struggle with adaptation due to their heightened sensitivity to social context and their reliance on external validation, making them more vulnerable to disorientation and stress in a new cultural environment (Berry et al., 1988; Mishra & Berry, 2017).

Empirical studies support these distinctions. Schmitz (1987; see also Schmitz & Schmitz, 2022) found that among Eastern European seasonal workers and other migrant groups in Germany, a moderate association existed between integration strategies and field independence. By contrast, field dependence was more commonly linked to separation strategies, and, to a lesser degree, assimilation, suggesting that cognitive style influences acculturation orientations.

Dogmatism—defined as a rigid, closed-minded cognitive stance (Rokeach, 1960)—also plays a role in shaping acculturation outcomes. High dogmatism has been associated with resistance to new cultural values and a tendency toward ethnocentrism. Studies on migrant populations (e.g., Schmitz, 1987; Taft & Steinkalk, 1985) suggest that lower dogmatism correlates inconsistently with integration and assimilation, while higher levels are more consistently linked to separation and marginalization (Schmitz & Berry, 2011). However, substantial variability across findings points to the complexity of this relationship and the potential moderating effects of other factors, such as cultural distance, education, or host country receptivity.

Sense-making refers to the cognitive–emotional process of deriving meaning from difficult or adverse life experiences. Within the framework of meaning-focused coping (Folkman & Moskowitz, 2000), sense-making enables individuals to cognitively reinterpret stressors, often through reframing negative experiences as growth opportunities or through attributing events to understandable causes (Davis et al., 1998; Holland et al., 2006). This cognitive reframing can be critical for migrants navigating the multifaceted stressors of resettlement, including loss, discrimination, and identity disruption.

A growing body of research supports the role of sense-making in facilitating positive psychological adaptation. Studies have shown that migrants who engage in sense-making—through acceptance, positive reinterpretation, or spiritual meaning—experience reduced stress, fewer depressive symptoms, and higher life satisfaction (Mykletun, 1985; Thompson, 1985). For instance, Ward et al. (1998) found that, among international students, coping strategies centered on growth and reinterpretation predicted lower stress levels. Similarly, Chataway and Berry (1989) observed that Chinese students in Canada who employed positive thinking reported greater satisfaction with their coping abilities. D. F. K. Wong (2002) found that Chinese immigrants in Hong Kong who viewed resettlement challenges as part of a broader journey of personal development showed more optimistic appraisals of their situation, suggesting that cognitive reframing can buffer the psychological impact of migration-related stress.

Meaning in life: Closely related to sense-making is the broader concept of meaning in life, which pertains to individuals’ perceptions of the purpose, coherence, and significance of their existence (Frankl, 1985; Steger & Frazier, 2005). The process of acculturation often prompts existential reflection, offering both challenges and opportunities for migrants to redefine their sense of identity and life goals (Pan, 2011; L. F. Wong, 2007).

Research consistently demonstrates that a strong sense of meaning in life enhances psychological well-being, resilience, and life satisfaction (Chamberlain & Zika, 1988; Mascaro & Rosen, 2005). Zika and Chamberlain (1987) identified meaning in life as the strongest predictor of positive emotions and life satisfaction among personal variables. Furthermore, meaning in life has been shown to mediate the effects of stress on well-being (Wallace & Lahti, 2004) and to mitigate the negative consequences of trauma (Krause, 2007). For migrants, a clear sense of life purpose can provide direction amidst cultural ambiguity, acting as a psychological anchor that fosters hope and emotional stability during difficult transitions.

In summary, cognitive characteristics such as cognitive style, sense-making, and meaning in life play a pivotal role in shaping how individuals experience and respond to acculturation. Field-independent individuals are more likely to navigate cultural transitions with confidence and maintain psychological stability, whereas those with field-dependent or dogmatic styles may face greater challenges due to heightened reliance on external validation and cognitive rigidity. The capacity to make sense of difficult experiences and derive meaning from them—whether through personal narratives, cultural reinterpretation, or spiritual beliefs—emerges as a crucial resource for psychological resilience and life satisfaction. These findings highlight the importance of cognitive flexibility and existential meaning in facilitating adaptive acculturation, and underscore the value of interventions that promote reflective thinking, value clarification, and cognitive reframing among migrant populations.

## 6. Identity and Acculturation

Ethnic identity and acculturation attitudes are often discussed in tandem in acculturation research, yet they are distinct constructs with different psychological underpinnings and developmental trajectories. Acculturation attitudes reflect an individual’s preferences and strategies for engaging with both their heritage and host cultures (e.g., integration, assimilation, separation, and marginalization (Berry, 1997)), while ethnic identity pertains to the subjective sense of belonging and emotional attachment to one’s ethnic group (Phinney, 1990, 2003; LaFromboise et al., 1993). Ethnic identity encompasses dimensions such as self-labeling, group commitment, shared values, and affective regard. Importantly, while both constructs are malleable, acculturation attitudes tend to be more flexible and contextually driven, whereas ethnic identity often develops more slowly and exhibits greater stability over time (Phinney, 1992).

Hutnik’s (1986, 1991) quadri-polar model offers a typological approach to ethnic self-identification among ethnic minorities in Britain, distinguishing four styles—Assimilative, Acculturative, Marginal, and Dissociative. This model usefully integrates dual identification with both the minority and majority cultures, reflecting the complexity of cultural adaptation processes. However, a critical limitation is that the model relies on self-reported identification patterns without robust empirical links to actual behavioral or psychological outcomes. For example, Hutnik found no significant relationship between ethnic identification style and cultural attitudes or behaviors among British Asians, raising questions about the predictive power and applicability of the framework across contexts and groups.

The alternation model proposed by LaFromboise et al. (1993) presents a more process-oriented perspective, emphasizing the ability to shift between cultural frames without compromising identity coherence. This model challenges the dichotomy of “either/or” identity frameworks by positing that individuals can maintain competence and authenticity in two cultures simultaneously. Similarly, Clément and Noels (1992) introduced the notion of ethnolinguistic identity fluidity, where identity shifts are situational and strategic. These approaches collectively critique rigid identity taxonomies, advocating instead for dynamic and contextualized understandings of cultural affiliation. Nevertheless, these models require further empirical validation, particularly in how they address power imbalances, discrimination, and identity threat in real-world inter-cultural contexts.

Phinney’s (1989, 1990) widely cited three-stage model of ethnic identity development—comprising unexamined identity, exploration, and achieved identity—provides a developmental framework that integrates psychosocial and cultural dimensions. Drawing from earlier models (e.g., Cross’s Nigrescence model), Phinney’s approach emphasizes that identity is not static but evolves through experiences, especially encounters with prejudice or cultural dissonance. However, critics argue that this model may inadequately capture non-linear or cyclical patterns of identity development, particularly for individuals in multi-cultural or globalized environments where identity negotiation is ongoing rather than sequential. Moreover, Phinney’s model, while influential, is often criticized for its reliance on self-report scales that may fail to fully reflect the nuanced emotional and cognitive processes of ethnic identity formation (Syed & Azmitia, 2008).

A growing body of research has focused on Bicultural Identity Integration (BII), which reflects how individuals cognitively and affectively perceive the compatibility of their dual cultural identities. High-BII individuals perceive their cultural backgrounds as harmonious and mutually enriching, leading to greater psychological well-being, lower acculturative stress, and enhanced cognitive flexibility and complexity (Benet-Martínez & Haritatos, 2005; Benet-Martínez et al., 2006; Berry et al., 2006; Nguyen & Benet-Martínez, 2013). In contrast, low BII is associated with identity conflict, reduced cultural competence, and impaired psychological functioning. Importantly, BII is not merely a personal trait but is shaped by structural and contextual factors, such as perceived discrimination, societal inclusiveness, and intergroup attitudes. As such, promoting high BII may require systemic interventions that foster inter-cultural openness and reduce ethnocentric norms in host societies.

Phinney and Devich-Navarro’s (1997) classification of biculturals into blended, alternating, and separated categories parallels Berry’s acculturation typology, offering a more fine-grained look at identity outcomes. Research suggests that blended biculturals—who fuse aspects of both cultures into a coherent whole—tend to exhibit better psychological adaptation than alternating biculturals, who compartmentalize their identities depending on context (Ward et al., 2018). However, such typologies risk oversimplifying the lived experiences of biculturals, who may shift between identity strategies depending on relational dynamics, societal feedback, and life-stage transitions. Furthermore, the idea that blended identities are inherently “better adjusted” may inadvertently pathologize legitimate forms of compartmentalization or strategic code-switching used by individuals navigating exclusionary environments.

Critically, most identity models in acculturation research have been developed within Western individualist frameworks, potentially limiting their cultural applicability and relevance across collectivist or non-Western settings (J. Schwartz et al., 2010). Moreover, the heavy focus on psychological adjustment risks overlooking sociopolitical dimensions of identity, such as resistance, cultural activism, and collective memory, which play a vital role in identity formation among diasporic and racialized communities.

In sum, identity and acculturation are distinct but interwoven constructs that shape how individuals navigate inter-cultural contact. While ethnic identity reflects deep-rooted psychological and affective bonds with one’s cultural group, acculturation attitudes express more fluid, context-dependent preferences about cultural engagement. Models such as Hutnik’s typology, Phinney’s developmental framework, and BII theory offer valuable insights but also face limitations related to empirical consistency, cultural bias, and insufficient attention to structural context. Future research should move beyond static classifications toward process-oriented, intersectional, and context-sensitive models that capture the complexity of identity formation in an increasingly multi-cultural world. Understanding these nuances is essential for developing inclusive social policies, educational interventions, and clinical practices that support positive identity development and inter-cultural competence.

## 7. Discussion

The literature reviewed underscores the growing consensus that acculturation is a dynamic, context-dependent process, shaped not only by stable personality traits but by their interaction with social, cultural, and structural factors. While early models of acculturation emphasized predefined strategies (Berry, 1997), this body of research supports a shift toward integrative frameworks that acknowledge the interplay of personality, motivation, emotions, culture, and social environment in shaping migrants’ psychological and socio-cultural adaptation.

A key contribution of the reviewed findings is the validation of the cultural fit hypothesis (Searle & Ward, 1990), which argues that the success of acculturation is contingent on the compatibility between an individual’s psychological traits and the normative expectations of the host culture. Traits such as extraversion, while generally associated with positive outcomes in individualistic societies, may lead to conflict or marginalization in collectivist settings where restraint and indirectness are valued. This suggests that trait adaptiveness is not fixed but culturally relative, challenging universalist assumptions in earlier trait-based models.

In addition, the findings highlight that acculturation is not solely a function of personality or choice, but is shaped by external conditions such as racism, discrimination, legal status, and access to social support. These insights reinforce the need to move beyond individual-centric frameworks toward ecological and intersectional approaches, where psychological processes are understood in relation to broader sociopolitical realities. This aligns with emerging trends in acculturation research that stress the importance of power, status, inequality, and systemic barriers in shaping adaptation outcomes.

Moreover, the recognition of motivational factors, such as intrinsic motivation, promotion focus, and the need for cognitive closure, provides a more nuanced understanding of the psychological drivers behind acculturation strategies. Migrants with proactive, hope-oriented mindsets may be better equipped to navigate cultural challenges, particularly when these motivations are supported by structural opportunities for engagement and growth. These aspects also open space for investigating less traditional personality constructs—such as optimism, self-monitoring, or meaning-making—that have been under-represented in the acculturation literature but appear to play a significant role in adaptation and well-being.

The reviewed research further expands the scope of acculturation theory by emphasizing cognitive and existential dimensions, such as cognitive style, reflective thinking, and the search for meaning. The ability to reframe negative experiences and derive personal meaning from cultural transition emerges as a critical resource for resilience and psychological adjustment. This approach resonates with eudaimonic models of well-being (Ryff & Singer, 2006), which foreground personal growth, autonomy, and life purpose as key indicators of adaptation, moving the field beyond narrow definitions of stress reduction or functional integration.

A key limitation of the manuscript is its predominant reliance on research conducted within Western contexts, which significantly constrains the global applicability and cultural sensitivity of its findings. Much of the existing literature assumes a Western-centric framework of acculturation that fails to account for the vast heterogeneity of migration experiences across the globe. Scholars such as Bhatia and Ram (2009) have argued that dominant acculturation models are rooted in Enlightenment-era, individualistic assumptions that universalize the migrant experience, thereby erasing the structural inequalities, colonial histories, and geopolitical forces that disproportionately affect migrants from formerly colonized or politically unstable regions. These critiques underscore how acculturation cannot be divorced from global power relations, as migrants’ opportunities for adaptation are often shaped by legacies of imperialism, racial hierarchies, and sociopolitical marginalization. Cheng et al. (2016) extend this critique by showing that the dynamics of acculturation vary greatly depending on the host country’s colonial past, immigration policies, and prevailing ideologies about race and belonging. For example, migrants from former colonies may carry a unique historical consciousness or expectation of cultural familiarity that complicates their acculturative experiences in ex-colonial powers (Cheng et al., 2016). Furthermore, Rudmin (2009) highlights that most acculturation research disproportionately focuses on voluntary, economically motivated migrants to Western countries—such as international students or skilled workers—while neglecting forced migrants such as refugees, asylum seekers, and internally displaced persons, whose psychological and cultural adaptations are often shaped by trauma, coercion, and legal precarity. These gaps limit the theoretical comprehensiveness of acculturation research and raise ethical concerns about whose experiences are prioritized or ignored. As such, there is an urgent need for the field to incorporate intersectional, decolonial, and context-sensitive approaches that recognize the diverse pathways through which individuals and groups engage with cultural change, particularly in non-Western and structurally marginalized contexts.

The refined understanding of acculturation has direct implications for intervention design and policy development:

Culturally Tailored Interventions: Psychological interventions should avoid one-size-fits-all models and instead be adapted to the cultural context of both the migrant and the host society. Programs that teach cultural norms, communication styles, and context-appropriate behaviors—while also validating migrants’ heritage identities—can foster greater inter-cultural competence and self-efficacy.

Trait–Context Matching: Assessment tools and counseling strategies should consider not just individual traits but how these align or clash with the host culture’s social norms. For instance, migrants high in extraversion may benefit from coaching in culturally sensitive social skills, while those high in neuroticism might require enhanced emotional regulation training in high-stress environments.

Promotion of Meaning and Purpose: Therapeutic approaches that incorporate narrative therapy, value clarification, and existential reflection can support migrants in constructing coherent self-concepts and finding meaning in the migration journey, thereby enhancing psychological resilience.

Motivational Support Programs: Programs that cultivate intrinsic motivation, hope, and goal-setting can improve adaptive engagement with a host culture. Motivational Interviewing (MI) techniques, for example, could be integrated into migrant support services to boost agency and personal investment in the acculturation process.

Structural Interventions and Policy Reform: At the policy level, creating environments that reduce structural barriers (e.g., legal discrimination, limited access to services, and language marginalization) is essential. Integration policies should not only provide resources (education, housing, and employment) but also affirm cultural pluralism and social inclusion, which can reduce acculturative stress and improve outcomes.

Longitudinal and Context-Sensitive Research: Future research should prioritize longitudinal designs to track how psychological traits, motivations, and coping strategies evolve over time and in response to changing conditions. Mixed-method approaches that include qualitative insights from migrants can deepen our understanding of how meaning-making and cognitive styles influence adaptation.

## 8. General Conclusions

Although extensive research has examined the relationship between personality traits and acculturation, consistent findings remain limited, particularly outside traits like neuroticism. More importantly, the field is shifting toward an understanding of acculturation as a dynamic, transactional process, influenced by the interaction between individual dispositions and socio-cultural context. Models such as the cultural fit hypothesis provide a more accurate and context-sensitive framework for understanding migrant adaptation. Additionally, expanding the focus beyond the Big Five to include motivational, cognitive, and existential dimensions offers a more holistic understanding of migrant well-being. Psychological and policy interventions must reflect this complexity by being ecologically informed, culturally responsive, and developmentally attuned to the lived experiences of diverse migrant populations.
